# Effects of Fipronil Exposure on Glucose Metabolism Disorder via the Gut Microbiota and Inflammation

**DOI:** 10.3390/toxics14030207

**Published:** 2026-02-27

**Authors:** Ziquan Lv, Yuxuan Wu, Tingting Cao, Changfeng Peng, Xuan Zou, Xinyue Xu, Dan Wang, Ying Chen, Guangnan Liu, Yuebin Ke, Suli Huang, Yajie Guo

**Affiliations:** 1 School of Public Health, University of South China, Hengyang 421001, China; lvziquan1984@126.com (Z.L.); wyx110900@163.com (Y.W.); 2 Division of Conservation and Application of Biological Resources, Shenzhen Center for Disease Control and Prevention, Shenzhen 518055, China; caotting@163.com (T.C.); changfengpeng1993@163.com (C.P.); xuanzou2024@163.com (X.Z.); xuxinyue19992022@163.com (X.X.); wangdan_12021@163.com (D.W.); yiris96@126.com (Y.C.); liugn1022@163.com (G.L.); keyke@szu.edu.cn (Y.K.); 3 School of Public Health, Shenzhen University Medical School, Shenzhen University, Shenzhen 518055, China; 4 College of Animal Science, Anhui Science and Technology University, Chuzhou 233100, China

**Keywords:** fipronil, glucose metabolism, gut microbiota, bile acid, inflammation

## Abstract

Fipronil (FPN), a widely used insecticide, poses health risks through environmental contamination. Although its toxicity is increasingly recognized, the impact of fipronil on glucose metabolism remains poorly understood. In this study, mice on a normal diet (ND) or high-fat diet (HFD) received a daily oral administration of fipronil (0, 0.25, 1, or 4 mg/kg) for 35 days. Blood glucose and insulin were measured, and glucose/insulin/pyruvate tolerance tests were performed. We found that fipronil compromised glucose tolerance in mice fed an ND. Gut microbiota composition was assessed by 16S rRNA sequencing and the expression of inflammatory factors was detected in the tissues. Serum fibroblast growth factor 15 (FGF15) and bile acid were determined. In HFD-fed mice, fipronil exacerbated glucose metabolic disorders and enhanced insulin resistance. These metabolic disturbances were associated with gut microbiota dysbiosis, particularly a marked reduction in *Akkermansia muciniphila* (*A. muciniphila*) abundance, and increased systemic inflammation. Fipronil exposure also decreased serum FGF15 and elevated serum bile acids. Our results suggest that fipronil disrupts glucose metabolism in association with gut microbiota alterations, impairment of the FGF15-bile acid axis, and induction of inflammation, highlighting its potential relevance to diabetes risk. Further studies are warranted to validate our findings.

## 1. Introduction

Diabetes constitutes a significant cause of mortality, morbidity, and imposes substantial costs on health systems across the globe [1]. Type 2 diabetes mellitus (T2DM), encompassing roughly 90% of global diabetes cases, is distinguished by impaired glucose homeostasis [2]. The rising global burden of T2DM now constitutes a significant public health challenge. While genetic factors and alterations in lifestyle are key factors influencing the development of metabolic syndromes, environmental pollutant exposure has also been suspected to play a crucial role [3].

Categorized as a phenylpyrazole insecticide, fipronil (FPN) is extensively utilized for its systemic activity and broad-spectrum efficacy against diverse insect pests. It is primarily employed to combat pests in residential areas, veterinary settings, and agricultural fields [4]. In the environment, fipronil can form metabolites such as fipronil sulfide, fipronil sulfone, fipronil amide, or fipronil desulfuryl. These metabolites may exhibit higher or lower toxicity and persistence than the parent compound [5]. Monitoring efforts have confirmed the presence of fipronil and its degradation products in a wide array of environmental media. For instance, in the California River water samples, median fipronil levels was found to range from 204 ng/L to 440 ng/L [6]. In Europe, the residual fipronil concentration in the rivers has witnessed an increase in recent years [7]. In the Chinese adult population, the primary contributor to dietary fipronil intake is eggs (55.3%), while vegetables, meat, grains and the other food contribute approximately 30.7%, 5.90%, 5.30% and less than 2%, respectively [8]. These studies have validated the substantial presence of fipronil in the environment and food, thereby leading to an elevated exposure risk.

Fipronil exerts adverse effects on multiple organ systems. Its primary insecticidal action stems from the antagonism of γ-aminobutyric acid (GABA)-gated chloride channels, leading to neuronal hyperexcitation [4]. In mammals, while its acute neurotoxicity is lower due to species-selective receptor affinity, chronic exposure can induce toxicity through alternative pathways. Notably, fipronil is metabolized primarily by cytochrome P450 enzymes (e.g., CYP3A4) in the liver, a process that generates reactive metabolites and induces significant oxidative stress, contributing to hepatotoxicity [9]. This oxidative damage is a pivotal mechanism underlying several of its subacute toxic effects, including the disruption of thyroid function and potential contributions to neurobehavioral alterations [10]. Furthermore, fipronil has been documented to cause genotoxicity, reproductive toxicity, and developmental toxicity [11,12,13]. Notably, preliminary evidence suggests potential metabolic effects: the fipronil-treated group exhibited a significant decrease in glucose concentration in carp, while tadpoles demonstrated oxidative stress [14,15]. However, systematic research concerning how fipronil influences glucose metabolism and its underlying mechanisms is still lacking. While exposure to pesticides like chlorpyrifos is known to disrupt glucose homeostasis via gut microbiota-mediated pathways [16], the specific mechanisms of fipronil, and whether they involve similar axes, remain to be elucidated.

Numerous investigations have established the critical involvement of gut microbiota and their derived metabolites in the pathogenesis of chronic metabolic conditions, including obesity, diabetes, and non-alcoholic fatty liver disease (NAFLD) [17,18]. Exposure to xenobiotics—including airborne pollutants and pesticides—has been increasingly linked to impaired glucose homeostasis, an effect thought to be mediated through alterations in gut microbial composition [19,20]. Intestinal microbiota are known to regulate host metabolism through multiple mechanisms, one of which involves the bile acid signaling pathway [21]. In T2DM, bile undergoes changes in both its quantity and composition—alterations that have been associated with acid metabolism [22]. The G protein-coupled bile acid receptor 1 and the nuclear receptor farnesoid X receptor (FXR) are key mediators of these effects, functioning as critical regulators of bile acid, lipid, glucose, and energy homeostasis. These intestinal receptors have garnered increasing research interest for their involvement in energy metabolism [23]. Upon activation by bile acids, ileal FXR induces the production of fibroblast growth factor 15/19 (FGF15/19), an atypical FGF that functions as an enterokine. This hormone then travels to the liver, where it binds to receptor complexes on hepatocytes to suppress bile acid synthesis and gluconeogenesis while promoting glycogen and protein synthesis [24]. 

While concerns exist regarding the human health effects of fipronil exposure, and the interrelationship among gut microbiota, inflammation, and glucose metabolism is recognized, the potential role of fipronil in modulating this interplay remains poorly understood. In this study, we investigated the effects of different doses of fipronil on glucose metabolism in mice fed with the normal diet or high-fat diet patterns. We hypothesized that fipronil exposure can disrupt glucose metabolism by disrupting the FGF15–bile acid axis (via reduction in gut microbiota) and triggering inflammation, ultimately leading to insulin resistance and increased gluconeogenesis. This study investigated the risk of fipronil-induced T2DM and elucidates the underlying mechanisms, with the ultimate goal of providing scientific guidance for the prevention, control, and intervention of T2DM.

## 2. Materials and Methods

### 2.1. Materials

Fipronil (≥98%, F110005) and pure corn oil (100%, C116023) were sourced from Sigma-Aldrich(St. Louis, MO, USA). The mice were fed either a high fat diet (HFD; 60 kcal% fat, D12492), or normal diet (ND; 10 kcal% fat, D12450), both from Research Diets Inc. (New Brunswick, NJ, USA).

### 2.2. Animals and Treatments

Fifty-six male C57BL/6J mice aged 7–8 weeks were obtained from the Guangdong Medical Laboratory Animal Center (Guangdong, China). Throughout the experiment, animals were kept under standard laboratory conditions (22–25 °C ambient temperature; 12-h light/dark photoperiod). Food and water were available ad libitum, except when temporary deprivation was mandated by experimental protocols. The Institutional Animal Care and Use Committee of the Shenzhen Center for Disease Control and Prevention reviewed and authorized all experimental protocols involving animals (GB/T 35892-2018, no. 2020010) [25]. Animal experiments were executed following the principles set forth in the ARRIVE guidelines.

The mice were randomly divided using a random number table into four groups (control, 0.25 mg/kg fipronil, 1 mg/kg fipronil and 4 mg/kg fipronil) with seven mice in each group, and fed with either ND or HFD. A sample size of seven mice per group was chosen based on common practice in comparable murine metabolic and toxicological studies (typically 6–8 animals per group) [24]. The mice were treated with fipronil or corn oil at different doses by oral gavage, administered daily for five weeks. Data were collected and analyzed by investigators unaware of treatment group identities. Blood glucose analyses were carried out in the fifth week of the experimental period. Cecal contents of each mouse were collected in the fifth week. Predefined exclusion criteria (e.g., severe injury or technical failure) and humane endpoints (including sustained weight loss > 20% or severe lethargy) were established; no animals met these criteria during the study.

### 2.3. Blood Glucose and Serum Insulin Concentrations, Homeostatic Model Assessment of Insulin Resistance (HOMA-IR) Index

Blood glucose concentrations were determined using a FreeStyle Optium Neo meter (Abbott, Shanghai, China). Serum insulin concentrations were measured using a mouse Ultrasensitive Insulin ELISA Kit (80-INSHU-E01, YS00023, ALPCO, Salem, NH, USA). The HOMA-IR index score was calculated according to the formula described previously [26]: [fasting glucose levels (mmol/L) × [fasting serum insulin (μU/mL)]/22.5.

### 2.4. Glucose Tolerance Test (GTT), Insulin Tolerance Test (ITT), and Pyruvate Tolerance Tests (PTT)

To access the impact of fipronil on glucose homeostasis, the GTT, ITT, and PTT were performed on conscious mice. For GTT, animals were fasted for 12 h prior to the intraperitoneal (i.p.) injection of D-glucose (2 g/kg). For PTT, a 16-h fasting period preceded pyruvate injection. ITT was conducted after a 4-h fast, with i.p. administration of insulin (0.75 U/kg). Tail blood samples obtained at 0, 15, 30, 60, 90, and 120 min were assayed for glucose levels.

### 2.5. Liver Glycogen Assay

Following excision, liver tissue was immediately frozen via liquid nitrogen immersion and subsequently held at −80 °C pending glycogen determination. The tissue was immersed in Na_2_SO_4_ saturated with 30% KOH, then heated in a dry bath heater at 100 °C for 20 min. After the tissue was completely dissolved, 95% ethanol was added, and the mixture was frozen for 30 min. After centrifugation, glycogen was precipitated. Hepatic glycogen concentrations were determined by acid hydrolysis [27].

### 2.6. In Vivo Insulin Signaling

After a 4-h fast, anesthetized mice received an intraportal insulin injection (2 U/kg). At 3, 4, and 5 min after injection, the liver, white adipose tissue (WAT) and soleus muscle samples were collected.

### 2.7. FGF15 and Bile Acid Measurement

Serum samples were collected and stored at −80 °C until analysis. A mouse FGF15 ELISA Kit (MEL154Mu, Wuhan USCN Commerce Co., Ltd., Wuhan, China) was used to determine the concentration of mouse FGF15 in serum. Bile acid was then measured using a total bile acid assay kit (Colorimetric, Sigma-Aldrich, St. Louis, MO, USA).

### 2.8. Total Protein Extraction and Western Blot

Protein lysates were prepared by homogenizing tissues in radioimmunoprecipitation assay buffer solution (Thermo Fisher Scientific, Waltham, MA, USA). Electrophoretic separation was performed on 10% sodium dodecyl sulfate–polyacrylamide gel electrophoresis (SDS-PAGE), followed by transfer to polyvinylidene fluoride (PVDF) membrane. After blocking with 5% skimmed milk for 1 h at room temperature, the membranes were probed with primary antibody overnight at 4 °C, followed by incubation with the secondary antibody for two hours. Finally, the signal was detected using an Odyssey infra-red imaging system (Thermo Fisher Scientific, Waltham, MA, USA). The housekeeping proteins tubulin or β-actin were used to standardize the protein density.

Primary antibodies used for Western blotting included: anti-p-IR (Tyr1150/1151) (3024S), anti-IR (3025S), anti-p-AKT (Ser473) (9271S), anti-AKT (9272S), anti-p-GSK-3β (Ser9) (9336S), anti-GSK-3β (9315S) anti-tubulin (3873S), anti-IL-6 (D5W4V) (12912S), anti-IL-1β (3A6) (12242S), anti-TNF-α (D2D4) (11948S), anti-NF-κB (C22B4) (66286SF) (all obtained from Cell Signaling Technology, Beverly, MA, USA); anti-Pepck (sc-271029, Santa Cruz Biotechnology Inc., Dallas, TX, USA); anti-G6pase (ab83690), anti-FGF19 (ab225942), anti-TLR4 (ab13867), (all obtained from Abcam, Cambridge, MA, USA), and anti-actin (A5316, Sigma-Aldrich, St. Louis, MO, USA).

### 2.9. RNA Extraction and Quantitative Real-Time Polymerase Chain Reaction (qRT-PCR) Analysis

TRIzol reagent (Thermo Fisher Scientific, Waltham, MA, USA) was used to isolate total RNA from frozen tissues, following the manufacturer’s protocol. cDNA was synthesized using a PrimeScriptTM RT Master Mix (TaKaRa, Beijing, China). SYBR Green Master Mix was used to perform qRT-PCR (ABI, Foster City, CA, USA). The relative expression of genes was normalized to GAPDH. The primer sequences of the targeted genes are described in Table S1.

### 2.10. Gut Microbiota Analysis

Genomic DNA was extracted from cecal contents using the QIAamp DNA Stool Kit (Qiagen, Gaithersburg, MD, USA). DNA purity and concentration were measured using the NanoDrop One spectrophotometer (Thermo Fisher Scientific, Waltham, MA, USA). Barcoded primers were employed in conjunction with TaKaRa Premix Taq^®^ Version 2.0 (TaKaRa Biotechnology Co., Dalian, China) to amplify the V4–V5 hypervariable region of the 16S rRNA gene via PCR. PCR products were quantified using GeneTools Analysis Software (Version 4.03.05.0, SynGene), pooled at equal mass, and then purified with the E.Z.N.A.^®^ Gel Extraction Kit (Omega, Norcross, GA, USA). The purified amplicons were eluted with TE buffer. Sequencing libraries were prepared using the NEBNext^®^ Ultra™ II DNA Library Prep Kit for Illumina^®^ (New England Biolabs, Ipswich, MA, USA) according to the manufacturer’s protocol, and paired-end sequencing (2 × 250 bp) was conducted on the Illumina Nova 6000 platform.

Raw sequencing reads were processed using fastp (version 0.14.1) to remove adapters, trim low-quality bases with a sliding window of 4 bp (quality threshold 20). Reads falling below the length or quality cutoffs were discarded. Paired-end reads were merged using USEARCH (version 7.1) with the following parameters: maximum mismatches ≤ 5 bp, minimum overlap ≥ 16 bp, and minimum alignment identity ≥ 90%. Chimera detection and removal were carried out using the UCHIME algorithm. Using the UPARSE pipeline, operational taxonomic units (OTUs) were delineated at a 97% sequence identity threshold. Representative sequences were taxonomically assigned by alignment against the Greengenes database (version 13.8).

### 2.11. Statistical Analysis

Values are expressed as mean ± SEM, with each group consisting of 7 biologically independent mice. For molecular assays (e.g., qPCR), measurements were performed in technical triplicate. We employed LEfSe analysis to detect differentially abundant taxa, combining Kruskal–Wallis tests for initial screening, Wilcoxon tests for pairwise consistency, and LDA for effect size quantification. Significance was defined by a log10 LDA score threshold of 2.0. Alpha diversity was evaluated with the Chao1 index, and β-diversity was evaluated through principal coordinate analysis (PCoA) ordination derived from Bray–Curtis dissimilarity matrices. Taxa with an average relative abundance below 1% were excluded from visualization. Data handling and figure preparation were handled with GraphPad Prism 8.0 and R 4.3.1; *p* < 0.05 marked the significance boundary.

## 3. Results

### 3.1. Fipronil Disrupts Glucose Tolerance and Insulin Signaling in ND-Fed Mice

**Figure 1:**
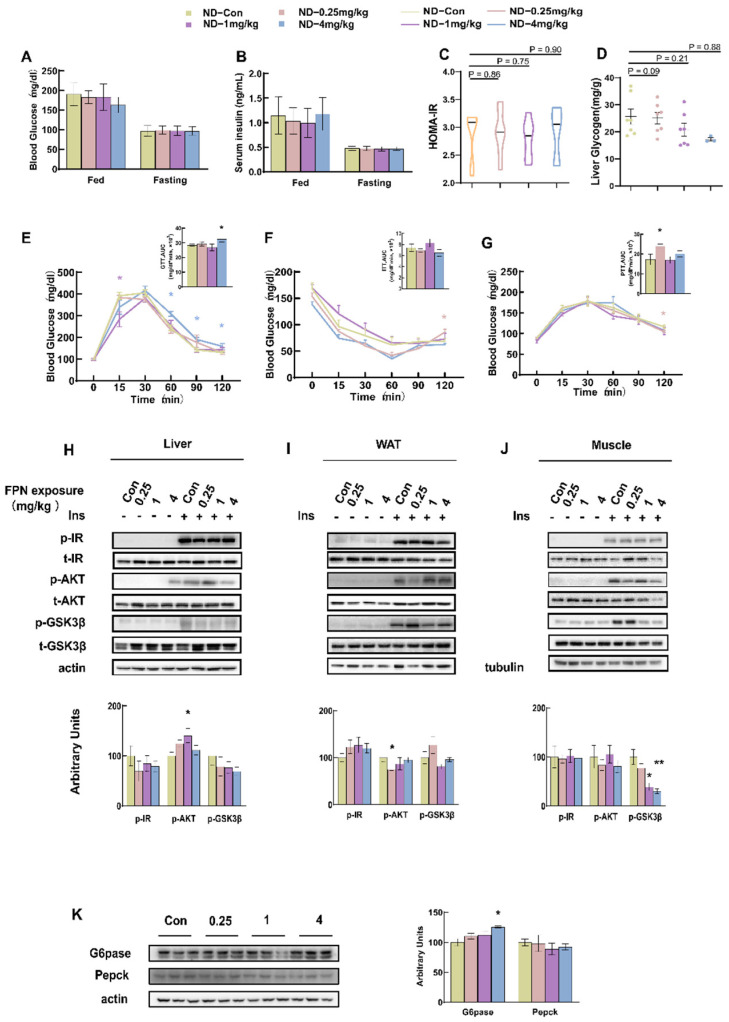
Glucose metabolic outcomes in ND mice following fipronil exposure. After 5 weeks of daily oral administration, glucose metabolic parameters were assessed in mice maintained on a normal diet. Measurements included the (**A**) blood glucose concentration (mg/dL), (**B**) serum insulin concentration (ng/mL), (**C**) HOMA-IR index, (**D**) liver glycogen (mg/g), (**E**) glucose tolerance test (GTT), (**F**) insulin tolerance test (ITT), and (**G**) pyruvate tolerance test (PTT). Mice were then sacrificed and tissues collected. (**H**) Phosphorylated (p-) and total (t-) IR, AKT, and GSK3β in the liver (loading control: β-actin). (**I**) Phosphorylated (p-) and total (t-) IR, AKT, and GSK3β in WAT (loading control: β-actin). (**J**) phosphorylated (p-) and total (t-) IR, AKT, and GSK3β in the soleus muscle (loading control: tubulin). (**K**) G6PASE and phosphoenolpyruvate carboxykinase (Pepck) proteins in the liver. Data are presented as mean ± SEMs, *n* = 7. *: *p* < 0.05, **: *p* < 0.01. Pairwise comparisons were made using the two-tailed unpaired Student’s *t*-test. When comparing three or more groups, one-way ANOVA was first performed, followed by the Student–Newman–Keuls post hoc test to identify specific differences.

Glucose metabolic responses to fipronil were investigated in 7- to 8-week-old male C57BL/6J mice following 5 weeks of daily oral gavage with 0, 0.25, 1, or 4 mg/kg·bw. Blood glucose levels were then measured under both fed and fasting conditions. As shown in Figure 1A–D, no significant difference was detected in the glucose levels, serum insulin concentrations, HOMA-IR index, or the liver glycogen concentrations between the fipronil-treated and control groups. Compared with the control group, GTT showed that mice administered with 4 mg/kg fipronil had impaired glucose tolerance (Figure 1E). We also measured insulin sensitivity or gluconeogenesis by ITT or PTT, respectively. The increase in gluconeogenesis was only observed upon treatment with 0.25 mg/kg of fipronil (Figure 1F,G). To investigate whether the enhanced insulin sensitivity phenotype resulted from the alterations in insulin signal transduction, we assessed the phosphorylation status of the insulin receptor (IR), protein kinase B (AKT), and glycogen synthase kinase 3β (GSK-3β) in liver, WAT, and soleus muscle following insulin stimulation. The results showed that the expression of p-AKT in the liver of 1 mg/kg fipronil-treated ND mice was significantly elevated (Figure 1H, *p* < 0.05).

In contrast, the expression of p-AKT in WAT was significantly decreased in the 0.25 mg/kg dosage group (Figure 1I, *p* < 0.05), while no significant difference was observed in the muscle tissue (Figure 1J). The levels of p-GSK-3β were decreased in the muscle of the dosage groups with 1 mg/kg and 4 mg/kg respectively (Figure 1J). This indicates that the insulin signaling pathway was inhibited. In the liver, the expression of glucose 6-phosphatase (G6pase), which is a protein related to gluconeogenesis, was significantly higher in mice receiving 4 mg/kg fipronil (Figure 1K). This suggests that gluconeogenesis was strengthened.

### 3.2. Fipronil Exposure Impairs Glucose Metabolism and Significantly Inhibits the Expression of Insulin Signaling Pathway Components in HFD Mice

**Figure 2:**
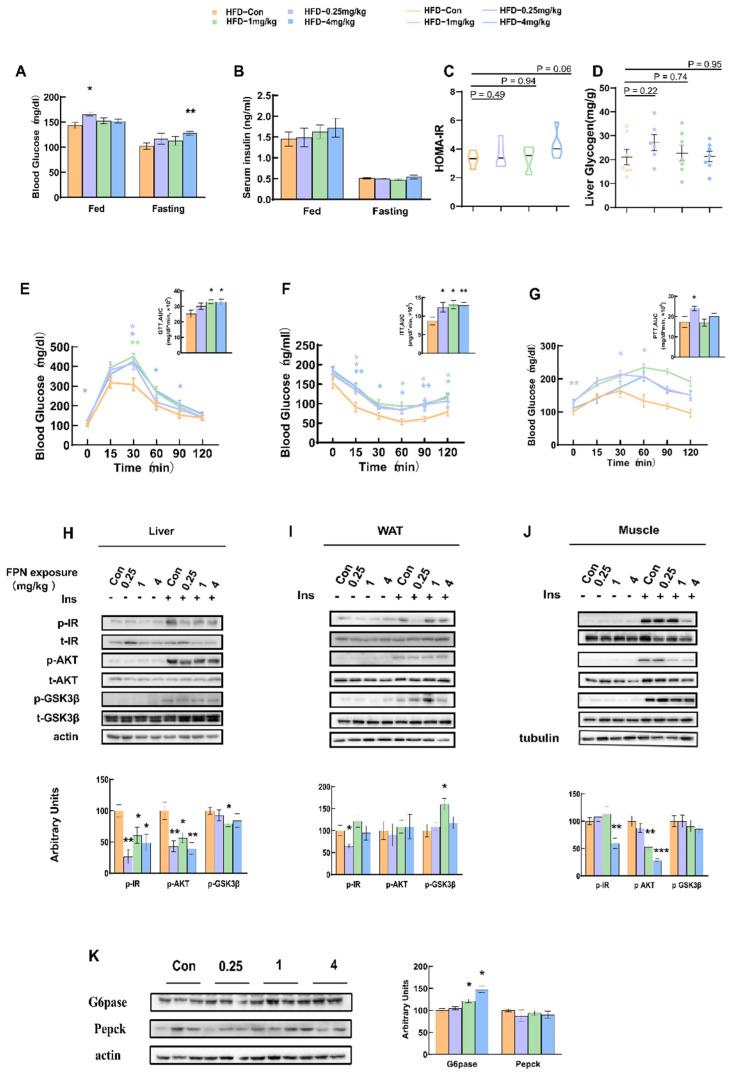
Glucose metabolic outcomes in HFD mice following fipronil exposure. After 5 weeks of daily oral administration, glucose metabolic parameters were assessed in mice maintained on a high-fat diet. Measurements included (**A**) blood glucose concentration (mg/dL), (**B**) serum insulin concentration (ng/mL), (**C**) HOMA-IR index, (**D**) liver glycogen (mg/g), (**E**) glucose tolerance test (GTT), (**F**) insulin tolerance test (ITT), and (**G**) pyruvate tolerance test (PTT). Mice were then sacrificed and tissues collected. (**H**) Phosphorylated (p-) and total (t-) IR, AKT, and GSK3β proteins in the liver (loading control: β-actin). (**I**) Phosphorylated (p-) and total (t-) IR, AKT, and GSK3β proteins in WAT (loading control: β-actin). (**J**) Phosphorylated (p-) and total (t-) IR, AKT, and GSK3β proteins in soleus muscle (loading control: tubulin). (**K**) G6PASE and Pepck proteins in liver. Data are presented as means ± SEMs, *n* = 7. *: *p* < 0.05, **: *p* < 0.01, ***: *p* < 0.001. Pairwise comparisons were made using the two-tailed unpaired Student’s *t*-test. When comparing three or more groups, one-way ANOVA was first performed, followed by the Student–Newman–Keuls post hoc test to identify specific differences.

We next examined the effects of fipronil in HFD-fed mice. After 5 weeks of oral gavage of fipronil, the HFD mice showed significantly impaired glucose metabolism. As shown in Figure 2A, mice receiving 0.25 mg/kg or 4 mg/kg fipronil exhibited notable elevations in blood glucose under both fed and fasting conditions (*p* < 0.05 or *p* < 0.01, respectively). There was no significant difference in the serum insulin concentrations during either the fed state or the fasting state among all the treated mice (Figure 2B). The HOMA-IR index was slightly increased in the mice treated with 4 mg/kg fipronil (*p* = 0.06, Figure 2C). No significant difference was found in the liver glycogen concentrations between the fipronil-treated and control groups (Figure 2D). Glucose tolerance was impaired in the mice treated with 1 mg/kg or 4 mg/kg fipronil (*p* < 0.05), while the insulin tolerance was impaired in all treated groups (*p* < 0.05), and the gluconeogenesis was significantly increased in the group treated with 0.25 mg/kg fipronil (*p* < 0.05, Figure 2E–G). This indicate that fipronil disrupted glucose metabolic homeostasis and induced hyperglycemia in the mice fed with HFD. Furthermore, an analysis of the levels of proteins in the key insulin signaling pathway within the liver revealed that the phosphorylated insulin receptor (p-IR) and phosphorylated protein kinase B (p-AKT) were significantly decreased in the groups treated with 0.25 mg/kg, 1 mg/kg or 4 mg/kg fipronil (*p* < 0.05, Figure 2H). p-GSK3β in the liver decreased significantly in the 1 mg/kg fipronil-treated group (*p* < 0.05), while no significant difference was found in the other groups (Figure 2H). The expression of GSK-3β in WAT increased in the 1 mg/kg fipronil-treated group (*p* < 0.05), and the level of p-IR decreased significantly in the 0.25 mg/kg fipronil-treated group (*p* < 0.05, Figure 2I). The levels of p-IR and p-AKT in the muscle of the 4 mg/kg fipronil-treated group were significantly reduced (*p* < 0.001), as was the level of p-AKT in the 1 mg/kg fipronil-treated group (*p* < 0.01, Figure 2J). Upon examining the expression of gluconeogenic proteins, we found that the expression of G6pase was significantly higher in the liver of the 1 mg/kg and 4 mg/kg fipronil-treated groups (*p* < 0.05) but did not observe a significant difference in phosphoenolpyruvate carboxykinase (Pepck) expression compared to the control group (Figure 2K).

### 3.3. Effects of Fipronil on the Gut Microbiota of ND Mice

**Figure 3:**
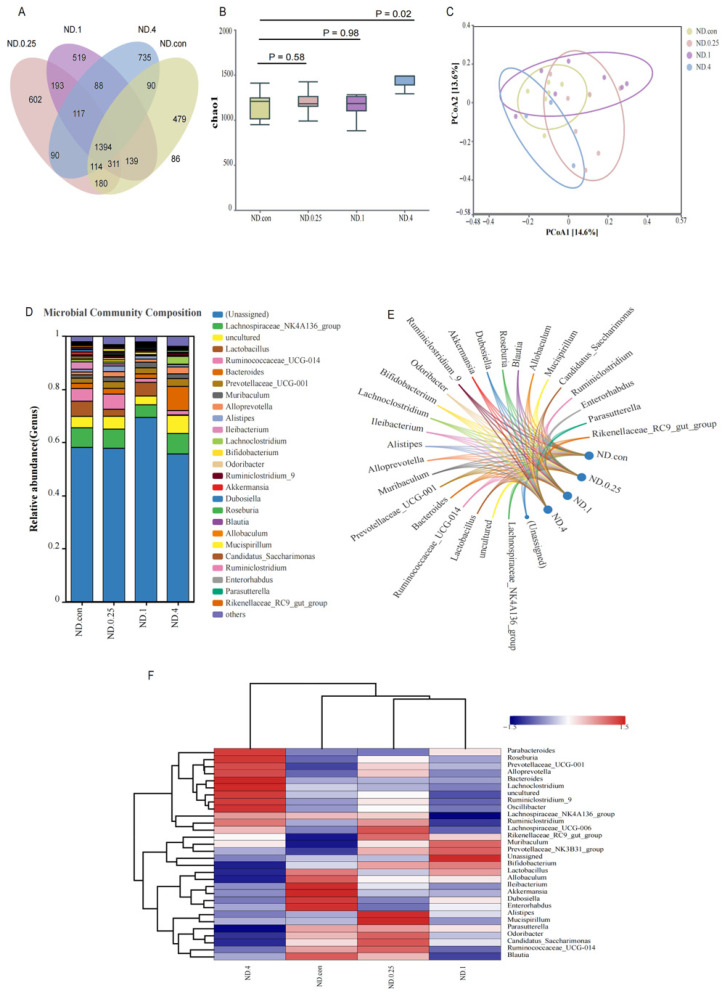
Fipronil-induced gut microbiota alterations in ND mice. (**A**) Venn diagrams. (**B**) α-Diversity based on Chao1 richness index. (**C**) Principal coordinate analysis (PCoA) ordination derived from Bray–Curtis dissimilarity matrices. (**D**) Relative abundances of the top 30 genus in each microcosm. (**E**) Chord diagrams depicting the distribution of dominant genus across groups. (**F**) Heatmap visualizing the abundance profiles of the top 30 genus. The color scale represents the row-normalized Z-score of relative abundance; red and blue indicate higher and lower abundance relative to the genus-specific mean, respectively. Horizontal represents grouping, vertical represents genus. *n* = 7.

16S rRNA gene sequencing was performed to investigate the cecal microbiota of mice treated with different doses of fipronil. The Venn diagram shows that 1394 OTUs were found to be shared among the four groups (Figure 3A). In addition, a significant difference was found in the microbial composition between the four groups. Specifically, four hundred and seventy-nine OTUs were unique to the ND control group, 602 OTUs were unique to the 0.25 mg/kg fipronil-treated group, 519 OTUs were unique to the 1 mg/kg fipronil-treated group, and 735 OTUs were unique to the 4 mg/kg fipronil-treated group (Figure 3A). The 4 mg/kg fipronil-treated group had a higher (*p* = 0.02) Chao1 index than the control group (Figure 3B). Beta diversity was determined by PCoA, which revealed that the cecal microbial communities of the four groups formed distinct clusters, as shown in Figure 3C. The fipronil-treated groups were clearly segregated, with PC1 and PC2 explaining 14.6% and 13.6% of the variance, respectively. At the genus level, taxa comprising more than 0.01% of the total gut microbial community per experimental condition were included in downstream analyses. Figure 3D,E shows the abundances of the top 30 bacterial genus. The Lachnospiraceae_NK4A136_group emerged as the dominant strain in the control (7.4%) and 0.25 mg/kg fipronil-treated group (6.7%) when we ignored the unassigned, uncultured, and others classifications. However, Lactobacillus was the dominant genus in the 1 mg/kg fipronil-treated group (5.2%), and Bacteroides was the dominant genus in the 4 mg/kg fipronil-treated group (9.1%). The individual treatment groups differed in composition at the genus level (Figure 3F). The heatmap showed that the bacteria of the fipronil-treated group formed a separate cluster from the control group. In the 4 mg/kg fipronil-treated group, genera including *Parabacteroides*, *Roseburia*, *Prevotellaceae_UCG-001*, *Alloprevotella*, *Bacteroides*, *Lachnoclostridium*, *Ruminiclostridium_9*, *Oscillibacter*, *Lachnospiraceae_NK4A136_group*, *Ruminiclostridium*, *Lachnospiraceae_UCG-006*, *Rikenellaceae_RC9_gut_group*, and *Muribaculum* were more abundant than in the controls, whereas *Allobaculum*, *Ileibacterium*, *Akkermansia*, *Dubosiella*, and *Enterorhabdus* were present at lower levels following fipronil treatment.

### 3.4. Effects of Fipronil on the Gut Microbiota of HFD Mice

**Figure 4:**
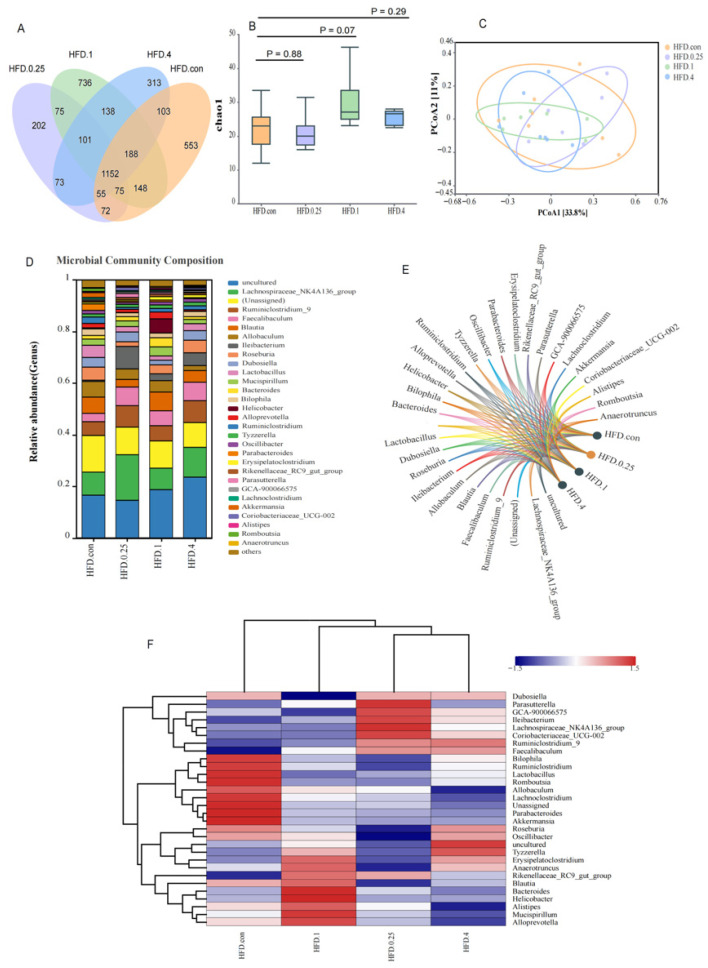
Fipronil-induced gut microbiota alterations in HFD mice. (**A**) Venn diagrams. (**B**) α-Diversity based on Chao1 richness index. (**C**) Principal coordinate analysis (PCoA) ordination derived from the Bray–Curtis dissimilarity matrices. (**D**) Relative abundances of the top 30 genus in each microcosm. (**E**) Chord diagrams depicting the distribution of dominant genus across groups. (**F**) Heatmap visualizing the abundance profiles of the top 30 genus. The color scale represents the row-normalized Z-score of relative abundance; red and blue indicate higher and lower abundance relative to the genus-specific mean, respectively. Horizontal represents grouping, vertical represents genus. *n* = 7.

We found 1152 OTUs to be shared among the four fipronil-treated groups (Figure 4A). Five hundred and fifty-three OTUs were unique to the HFD control group. In the fipronil-treated groups, the number of unique OTUs were 202 (0.25 mg/kg), 736 (1 mg/kg), and 313 (4 mg/kg). Compared with the HFD group, the unique OTUs of the 0.25 mg/kg and 4 mg/kg fipronil-treated group were significantly reduced. Analysis of the Chao1 index showed no significant difference in the α-diversity between the fipronil-treated and control groups (*p* > 0.05, Figure 4B). Beta diversity analysis showed distinct clustering of cecal microbial communities among the four fipronil-treated groups. The first two principal components captured 33.8% (PC1) and 11% (PC2) of the variation, effectively distinguishing the treatment groups (Figure 4C). The abundances of the top 30 bacterial genus in the HFD group are shown in Figure 4D,E. The *Lachnospiraceae_NK4A136_group*, with a prevalence ranging from 8.3% to 17.7%, emerged as the dominant one in all groups when we ignored the unassigned, uncultured, and others classifications.

Heatmap visualization demonstrated that fipronil treatment markedly altered the gut microbial landscape in HFD mice (Figure 4F). Relative to the controls, the fipronil-exposed samples exhibited decreased abundances of *Bilophila*, *Ruminiclostridium*, *Lactobacillus*, *Romboutsia*, *Allobaculum*, *Lachnoclostridium*, *Unassigned*, *Parabacteroides*, and *Akkermansia*, whereas *Ruminiclostridium_9*, *Faecalibaculum*, *Ileibacterium*, *Rikenellaceae_RC9_gut_group*, *Parasutterella*, and *Coriobacteriaceae_UCG-002* were increased.

### 3.5. Fipronil Decreases FGF15 Secretion and Elevates Serum Bile Acid Levels

**Figure 5:**
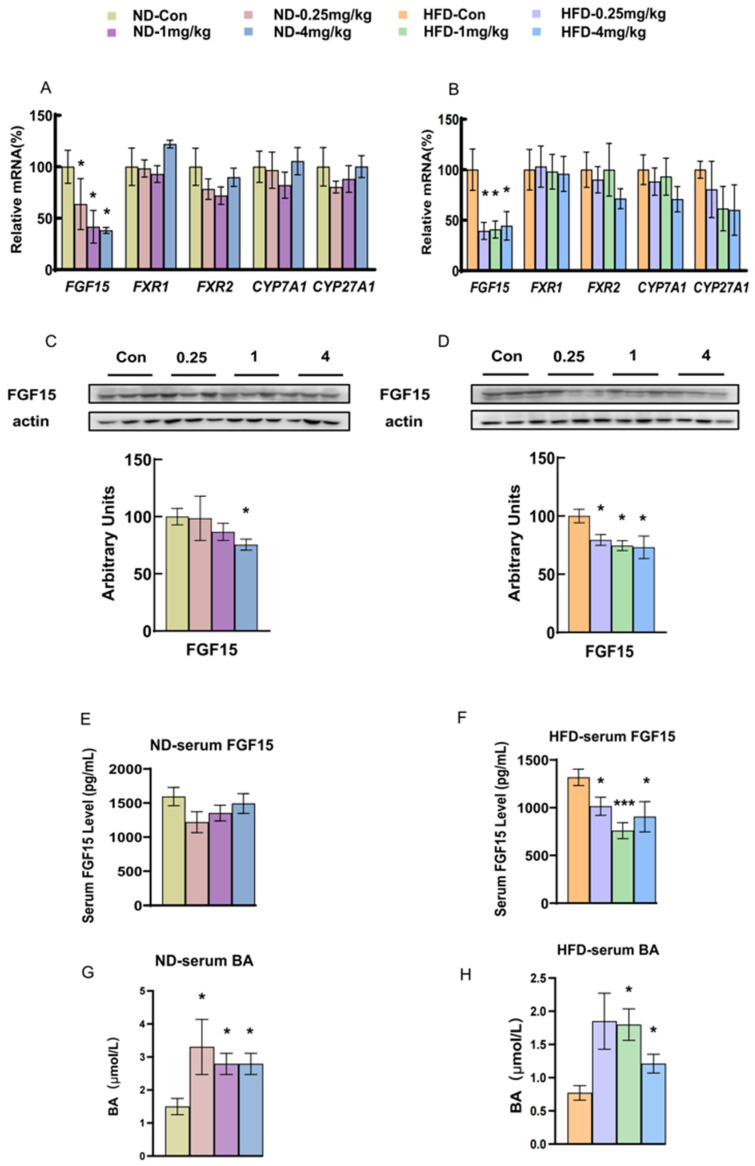
Fipronil has inhibitory effects on liver and serum FGF15, and increases serum bile acid concentrations. (**A**) Relative mRNA expression levels of liver FGF15, FXR1, FXR2, CYP7A1, and CYP27A1 in all groups of mice fed with ND. (**B**) Relative mRNA levels of liver FGF15, FXR1, FXR2, CYP7A1, and CYP27A1 in all groups of mice fed with HFD. (**C**) Relative protein levels of liver FGF15 in all groups of mice fed with ND. (**D**) Relative protein levels of liver FGF15 in all groups of mice fed with HFD. (**E**,**F**) Serum FGF15 levels in mice fed ND and HFD, respectively. (**G**,**H**) Serum total bile acid concentrations in ND and HFD mice. *n* = 7, *: *p* < 0.05, ***: *p* < 0.001. Pairwise comparisons were made using a two-tailed unpaired Student’s *t*-test. When comparing three or more groups, one-way ANOVA was first performed, followed by the Student–Newman–Keuls post hoc test to identify specific differences.

FGF15/19 is controlled by FGF activity and regulates the glycogen synthesis and cholesterol metabolism in the liver [28]. Fipronil reduced the expression of FGF15 in the liver in both the ND and HFD groups (*p* < 0.05, Figure 5A–D). However, no difference was observed in the expression of *CYP7A1*, *CYP27A1*, or FXR genes in the liver (Figure 5A,B). Next, we determined the serum concentrations of FGF15 and bile acid, and found that fipronil treatment decreased serum FGF15 in the mice fed with HFD, but not in the ND mice (Figure 5E,F). Mice exposed to fipronil exhibited significantly higher serum bile acid concentrations in both the ND and HFD groups (*p* < 0.05, Figure 5G,H).

### 3.6. Fipronil Induces Pro-Inflammatory Responses

**Figure 6:**
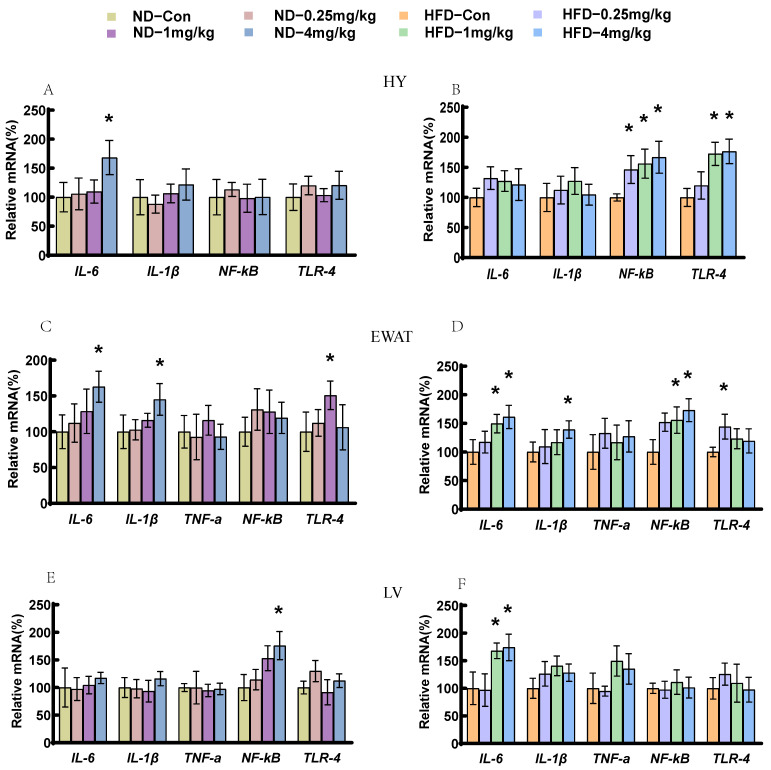
Fipronil promotes the gene expression of inflammatory factors in the hypothalamus, adipose tissue and liver. (**A**,**B**) IL-6, IL-1β, NF-κB, and TLR-4 mRNA levels in hypothalamus of ND (**A**) and HFD (**B**) mice. (**C**,**D**) IL-6, IL-1β, TNF-α, NF-κB, and TLR-4 mRNA levels in the EWAT of ND (**C**) and HFD (**D**) mice. (**E**,**F**) IL-6, IL-1β, NF-κB and TLR-4 mRNA levels in the liver of ND (**E**) and HFD (**F**) mice. (**F**) IL-6, IL-1β, TNF-α, NF-κB, and TLR-4 mRNA levels in the liver of HFD mice. *n* = 7, *: *p* < 0.05. Pairwise comparisons were made using the two-tailed unpaired Student’s *t*-test. When comparing three or more groups, one-way ANOVA was first performed, followed by the Student–Newman–Keuls post hoc test to identify specific differences.

To investigate whether fipronil induces inflammation, the expression of insulin resistance and obesity-related pro-inflammatory mediators was examined in the hypothalamus (HY) (interleukin [IL]-6, IL-1β, nuclear factor [NF]-κB), liver, and epididymal WAT (EWAT), as shown in Figure 6. In ND mice, the IL-6 mRNA levels were markedly higher in both HY and EWAT following 4 mg/kg fipronil treatment compared to the controls (*p* < 0.05, Figure 6A,C). IL-1β showed a significant rise only in EWAT at this dose, with no marked changes in other tissues (Figure 6C). Hepatic NF-κB expression was also upregulated exclusively at 4 mg/kg (*p* < 0.05, Figure 6E). In HFD mice, EWAT exhibited pronounced increases in IL-6 and NF-κB at both 1 and 4 mg/kg fipronil (*p* < 0.05), while IL-1β was elevated only at the highest dose (*p* < 0.05, Figure 6D). Hypothalamic NF-κB expression was significantly higher across all fipronil-treated groups (0.25, 1, and 4 mg/kg, *p* < 0.05, Figure 6B). Hepatic IL-6 levels were also substantially increased at 1 and 4 mg/kg (*p* < 0.05, Figure 6F). To investigate whether the TLR-4 pathway mediates fipronil-induced insulin resistance, we examined the expression of TLR-4 in the HY, EWAT, and liver. The expression of TLR-4 in EWAT in the ND mice increased in the 1 mg/kg fipronil-treated group (*p* < 0.05, Figure 6C). In the HFD mice, TLR-4 expression increased in the HY of the 1 mg/kg and 4 mg/kg fipronil-treated groups (*p* < 0.05, Figure 6B) and EWAT of the 0.25 mg/kg fipronil-treated group (*p* < 0.05, Figure 6D). Hepatic TLR-4 levels did not differ significantly following fipronil exposure (Figure 6F).

**Figure 7:**
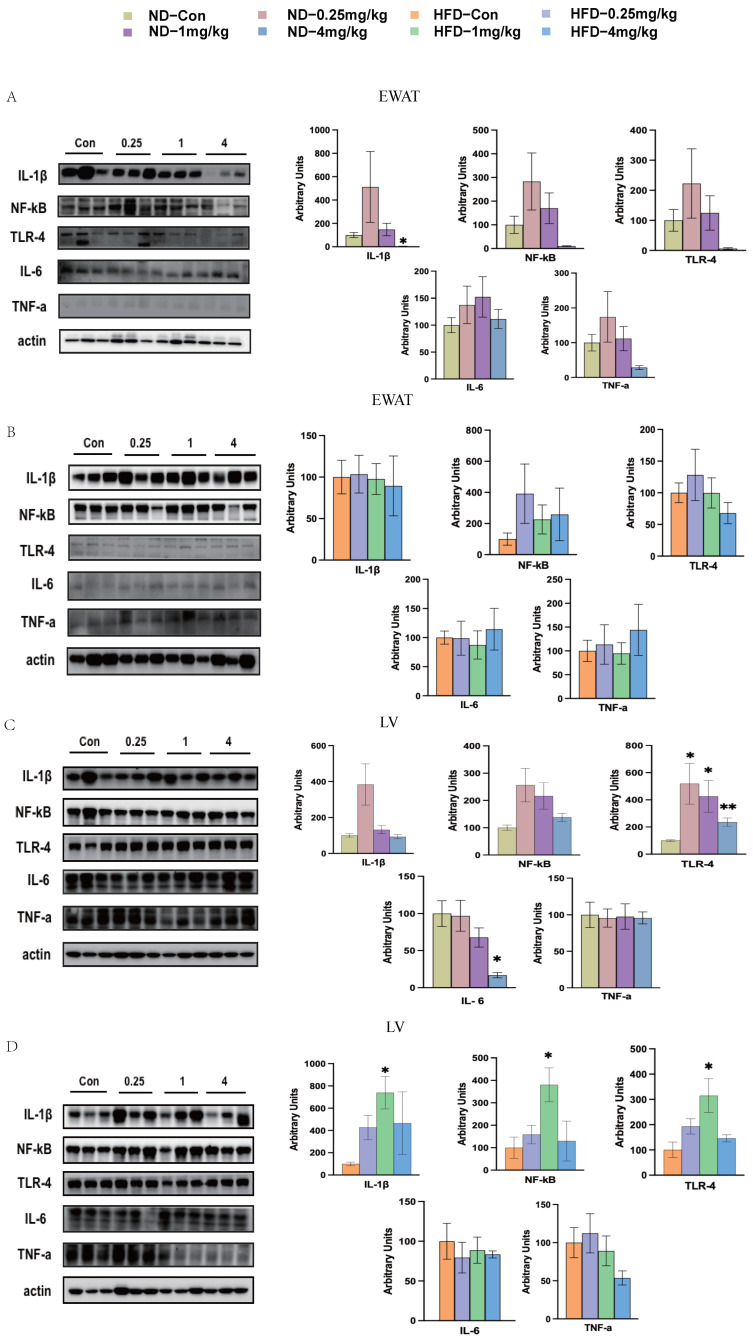
Fipronil regulates the protein levels of inflammatory factors in the adipose tissue and liver. (**A**,**B**) Protein levels of IL-1β, NF-κB, TLR-4, IL-6, and TNF-a in the EWAT of ND (**A**) and HFD (**B**) mice. (**C**,**D**) Protein levels of IL-1β, NF-κB, TLR-4, IL-6, and TNF-a in the liver of ND (**C**) and HFD (**D**) mice. *n* = 7, *: *p* < 0.05, **: *p* < 0.01. Pairwise comparisons were made using the two-tailed unpaired Student’s *t*-test. When comparing three or more groups, one-way ANOVA was first performed, followed by the Student–Newman–Keuls post hoc test to identify specific differences.

We profiled hepatic and EWAT protein expression in mice fed either diet (Figure 7). In EWAT, only IL-1β in the ND group was found to be decreased significantly at the 4 mg/kg dosage of fipronil treatment (*p* < 0.05, Figure 7A,B). We found that after fipronil treatment, the expressions of TLR-4 protein in liver at all dosages in the ND group were significantly increased compared with that of the control group, while the expression of IL-6 was significantly decreased only at 4 mg/kg (*p* < 0.05, Figure 7C). However, there was no difference in the expression of IL-1β, NF-κB, or TNF-a protein, consistent with their mRNA levels in the liver (Figure 6E and Figure 7C). The protein levels of IL-1β, NF-κB, and TLR-4 in the liver of HFD mice were significantly increased in the 1 mg/kg dosage group (*p* < 0.05, Figure 7D).

## 4. Discussion

As a commonly used pesticide in agriculture, the effect of fipronil exposure on human metabolic diseases and the molecular mechanisms remain poorly understood. Herein, we provide novel evidence regarding fipronil exposure and the disrupted glucose metabolism and insulin resistance. Fipronil-induced disruption of glucose metabolism was more pronounced in the HFD mice. Furthermore, our results demonstrate that fipronil exposure significantly reshaped the gut microbiota composition under both dietary conditions, with a consistent and notable decrease in the abundance of *Akkermansia*, *Allobaculum*, and *Lactobacillus*, alongside a notable elevation in *Rikenellaceae_RC9_gut_group*. Currently, studies have shown that *Akkermansia* is related to glucose metabolism [29]. Beneficial metabolic effects of *Akkermansia muciniphila* (*A. muciniphila)* administration in mice included enhanced glucose tolerance, reduced insulin resistance, and higher HDL cholesterol levels. [30]. Studies have shown that *A. muciniphila* prevents HFD-induced metabolic-associated fatty liver disease (MAFLD) by reducing FGF15-mediated intrahepatic bile acid synthesis [31]. Consistently, we found that the expression of FGF15 was inhibited, which was associated with a concomitant increase in systemic bile acid levels after fipronil exposure. Moreover, we found that fipronil may induce low-grade systemic inflammation in the HY, liver, and EWAT, resulting in insulin resistance. Collectively, our findings suggest that fipronil exposure impairs glucose metabolism and promotes insulin resistance through a mechanism involving gut microbiota-mediated disruption of the FGF15–bile acid axis and low-grade inflammation.

The fipronil dosages (0.25, 1, and 4 mg/kg·bw), which correspond to human equivalent doses of 0.03, 0.11, and 0.44 mg/kg·bw, respectively, were selected with consideration of reported human exposure levels in residential dust [32]. While these doses exceed typical environmental background concentrations, they are consistent with a mechanistic toxicology approach [33] and remain informative for specific high-exposure scenarios. For instance, fipronil residues in contaminated eggs reached 1.2 mg/kg during the 2017 European incident, and concentrations in residential dust have been reported up to 14.2 mg/kg [34]. Furthermore, ubiquitous human exposure to fipronil has been documented across diverse populations, including fipronil production workers (7.8 ng/mL in serum) [35], Korean males (0.130–3.570 ng/mL), and pregnant women (fipronil sulfone: 0.079–2.910 ng/mL) [36]. Collectively, these data establish that fipronil exposure in humans arises from dietary, environmental, and occupational sources, leading to widespread population exposure. Thus, although the present study does not replicate chronic low-level environmental exposure, it provides mechanistic insight into the metabolic disruption that may occur under elevated exposure conditions relevant to specific subpopulations and contamination events. Fipronil has been shown to induce hyperglycemia in carp [37] and European sea bass [38]. Fipronil can affect hepatic energy metabolism by activating glycogenolysis (glucose production) and glycolysis (lactic acid production from pyruvate). This metabolic disruption in the liver has been partly attributed to both the parent compound and its CYP450-generated metabolite fipronil sulfone, which impair mitochondrial function and disturb hepatic energy homeostasis [39]. This is consistent with our results. In addition, we also provide novel evidence that the magnitude of fipronil’s effect on glucose metabolism was influenced by the dietary context, with a more pronounced disruption effect observed in the HFD mice compared to mice fed with ND. However, although fipronil treatment had no effect on the blood glucose, insulin, HOMA-IR, or liver glycogen concentrations in ND mice, it significantly impaired glucose tolerance and increased gluconeogenesis, indicating that static glycemic parameters may be less sensitive than dynamic challenges in detecting early metabolic disturbances under normal dietary conditions. In HFD mice, fipronil significantly elevated fed and fasting glucose levels, whereas the HOMA-IR elevation was only marginal (*p* = 0.06 at 4 mg/kg). This trend, together with the clear impairment of insulin and glucose tolerance across multiple doses, suggests that fipronil exacerbates insulin resistance under metabolic stress, even though the HOMA-IR index may not fully capture the extent of the defect in this setting. Therefore, we conclude that fipronil affects glucose metabolism in mice irrespective of dietary context, though its impact is exacerbated under HFD conditions.

Mounting evidence points to a central part for the gut microbiome in the development of T2DM and insulin resistance [40,41]. Both diet and exposure to exogenous compounds can significantly influence gut microbial composition and function [42,43]. Indeed, we observed that fipronil affected gut microbiota composition in both ND and HFD mice. Notably, fipronil exposure resulted in a pronounced decrease in *A. muciniphila* counts relative to that observed in untreated mice. Depletion of *A. muciniphila* has been linked to multiple metabolic conditions, including obesity, T2DM, NAFLD, and cardiovascular diseases, in both rodent and human studies [29,44]. Moreover, supplementation with *A. muciniphila* alleviated the adverse metabolic consequences of a high-sugar diet in Caenorhabditis elegans, specifically blunting increases in both glucose and triglycerides [45]. Gut microbes influence host metabolism in part by modulating bile acid metabolism [46]. Bile acids play a key regulatory role in hepatic glucose and lipid metabolism, as evidenced by human and mouse studies; bile acid sequestrants are used clinically to lower cholesterol and improve metabolic parameters [47]. Intestinal administration of FXR agonists has also been shown to enhance TGR5 signaling and improve hepatic metabolism [48], underscoring the therapeutic potential of targeting bile acid pathways in metabolic diseases.

*A. muciniphila* has gained attention for its ability to ameliorate metabolic impairments, such as those heightened by environmental factors, positioning it as a promising next-generation probiotic. Specifically, *A. muciniphila* has been reported to counteract HFD-induced NAFLD via its actions on the enteric FXR–FGF15 axis and reshape the bile acid pool. This effect is mediated via the upregulation of FGF15, which suppresses bile acid synthesis [49]. Related studies indicate that *A. muciniphila* enhances insulin secretion and promotes FGF15/19 expression, leading to increased glycogen synthesis, the suppression of gluconeogenesis, and improved glucose tolerance [31]. Importantly, FGF19 (and its murine ortholog FGF15) exerts beneficial effects on glucose homeostasis through insulin-independent pathways [50,51]. In our study, fipronil exposure not only reduced the abundance of *A. muciniphila* but also decreased the serum FGF15 levels and increased the serum bile acids. This proposed mechanism is causally supported by studies showing that *A. muciniphila* supplementation rescues glucose intolerance, elevates FGF15/19, and suppresses bile acid synthesis in mice [52]. Additionally, evidence from other pesticides supports the involvement of gut microbiota in metabolic disruption. Using antibiotic treatment and fecal microbiota transplantation, Liang et al. demonstrated that chlorpyrifos-altered gut microbiota mediated the development of insulin resistance and obesity [16]. Based on these findings, we propose that fipronil reduces *A. muciniphila* abundance, leading to suppressed FGF15 expression. This suppression results in elevated serum bile acids, which in turn promotes hepatic gluconeogenesis and contributes to dysregulated glucose metabolism.

Insulin is a key hormone that regulates carbohydrate, lipid, and protein metabolism. Insulin resistance, a major driver of diabetes, is closely linked to chronic inflammation, which impairs insulin sensitivity [53]. Additionally, pesticide exposure was associated with major factors of T2DM, including adipose tissue inflammation and aberrant lipid deposition in the liver, muscle, and pancreas [54]; such exposure disrupts glucose homeostasis by promoting insulin resistance in adipocytes, thereby contributing to the dysregulation of lipid and glucose metabolism. [55]. Inflammation is now recognized as a core mechanism by which pesticides interfere with glucose metabolism. TNF-α and IL-1β, among other proinflammatory cytokines induced by various pesticides, activate NF-κB and JAK2/STAT3/SOCS3 signaling cascades, pathways also triggered by a high-fat diet, leading to impaired insulin action [20]. We reasoned that the development of insulin resistance following fipronil exposure may result from chronic, low-level activation of inflammatory signaling cascades. In our study, fipronil-treated mice displayed enhanced expression of multiple inflammatory markers—among them TNF-α, IL-6, IL-1β, NF-κB, and TLR-4—in the HY, EWAT, and liver, suggesting the presence of low-grade systemic inflammation in mice. This finding aligns with previous reports showing that fipronil triggers oxidative stress, inflammation, and apoptosis in adult zebrafish [13], and elevates levels of inflammatory mediators including IL-1β, IL-6, and IL-8 in human primary nasal epithelial cells [56]. Importantly, the causal role of inflammation in pesticide-induced metabolic dysfunction has been demonstrated in other models: chlorpyrifos exposure has been shown to trigger low-grade inflammation via gut barrier disruption and lipopolysaccharide translocation, and this inflammatory state was directly implicated in the onset of insulin resistance and obesity [16]. Parallel evidence from carbendazim, imidacloprid, diazinon, permethrin, and malathion further corroborates that pesticide-induced inflammation, accompanied by NF-κB activation and impaired glucose uptake, is a conserved mechanism preceding insulin resistance [20]. While our data revealed an association between fipronil-induced inflammation and impaired insulin signaling, the precise contribution of the observed inflammatory mediators—particularly the TLR4/NF-κB axis—remains correlative and requires direct experimental validation through targeted inhibition studies.

This study provides an initial, integrated exploration of how fipronil exposure may perturb glucose metabolism via gut microbiota-mediated FGF15–bile acid axis impairment and systemic inflammation. It provides new evidence and perspective for understanding how environmental pollutants contribute to the pathogenesis and progression of T2DM. A major strength of this research lies in its rigorous study design, which incorporated both normal and high-fat diet models, revealing the critical role of dietary context in its toxic effects. Furthermore, it employed a multi-level assessment spanning systemic glucose tolerance, tissue insulin signaling, and molecular gut microbiota/bile acid profiles, forming a integrated evidence chain. Several limitations of this study merit consideration. First, although we observed a consistent reduction in *A*. *muciniphila* accompanied by suppression of the FGF15–bile acid axis, the causal relationship between these events and fipronil-induced metabolic disturbances remains to be definitively established. Interventional approaches—such as fecal microbiota transplantation or *A. muciniphila* replenishment—would provide direct causal evidence, and the inclusion of a positive control group (e.g., mice colonized with a defined microbiota known to confer metabolic benefit) could further strengthen the specificity of the observed effects. Second, while our data indicate that fipronil-induced low-grade inflammation is associated with impaired insulin signaling, the precise molecular link remains correlative. Targeted inhibition of key inflammatory nodes (e.g., TLR4, NF-κB, or JAK2/STAT3) is required to determine whether these pathways play a necessary or sufficient role in driving insulin resistance upon fipronil exposure. Third, although the selected fipronil doses are grounded in realistic exposure data and are highly relevant for simulating specific exposure scenarios (e.g., through diet or household dust), the potential chronic health risks from long-term, low-dose environmental exposure across the general population warrant further investigation. Finally, although our findings are supported by mechanistic evidence from other pesticide classes and align with emerging human biomonitoring data, direct extrapolation to human health risk requires further investigation. Toxicokinetic modeling and well-designed epidemiological studies are needed to bridge the gap between subchronic rodent exposure and the chronic, low-level environmental intake experienced by human populations.

## 5. Conclusions

In summary, this study identifies fipronil as capable of disrupting glucose homeostasis through mechanisms involving gut microbiota dysbiosis, impairment of the FGF15–bile acid axis, and induction of systemic low-grade inflammation. While these pathways have been implicated in the metabolic toxicity of other pesticides, their operation in the context of fipronil exposure has not been previously demonstrated. Our findings further reveal that the metabolic effects of fipronil are more pronounced under high-fat diet conditions. This study provides a paradigm shift in assessing the health risks of agricultural contaminants. By uncovering a gut-microbiome mediated mechanism for fipronil-induced metabolic disruption, it supplies critical data for the early warning and risk assessment of chronic diseases like diabetes in exposed populations. The integrated approach developed here has promising applications in advancing environmental epidemiology, pollution source control, and public health intervention strategies.

## Data Availability

The original MiSeq 16S rRNA gene sequence data that support the mouse findings are available in the SRA under NCBI BioProject ID PRJNA894327, link: https://www.ncbi.nlm.nih.gov/bioproject?term=PRJNA894327&cmd=DetailsSearch (accessed on 23 January 2026).

## Supplementary Materials

The following supporting information can be downloaded at: https://www.mdpi.com/article/10.3390/toxics14030207/s1, Table S1: List of primer pairs for qRT-PCR; Figure S1: Experiment Design Flowchart.
