# Supplementary material for: Effects of Fipronil Exposure on Glucose Metabolism Disorder via the Gut Microbiota and Inflammation
Source: Toxics. 2026 Feb 27;14(3):207. doi: 10.3390/toxics14030207 (PMC13030138; doi:10.3390/toxics14030207)
Supplement: Supplementary file 1 [file toxics-14-00207-s001.zip › toxics-4131741-supplementary.pdf]

Supplementary Materials

Table S1. List of primer pairs for qRT-PCR.

Gene	Forward primer (5'–3')	Reverse primer (3'–5')
GAPDH	TGTGTCCGTCGTGGATCTGA	CCTGCTTCACCACCTTCTTGAT
FGF15	ATGGCGAGAAAGTGGAACGGGC	TTTCTGGAAGCTGGGACTC
FXR1	CCGATCGCATGGAGGAACTGACGGTGG	GTACTCCAGCAGCACCTGTACG
FXR2	TGTAAAGGATGTCCATGAAG	CTCTACTTCATCTCCTTCTG
CYP7A1	TCTGAACTTGATCACCGTCTCTCTG	GTCGACCAAATCTAGGCCAAAATCT
CYP27A1	CACGACATCCAACACGCTGAC	CCACAGGGTAGAGACGCAGAG
TNF-α	GGTGCCTATGTCTCAGCCTCTT	GCCATAGAACTGATGAGAGGGAG
IL-6	TCTATACCACTTCACAAGTCGGA	GAATTGCCATTGCACAACTCTTT
IL-1β	TGGACCTTCCAGGATGAGGACA	GTTCATCTCGGAGCCTGTAGTG
NF-κB	GCTGCCAAAGAAGGACACGACA	GGCAGGCTATTGCTCATCACAG
TLR-4	AGCTTCTCCAATTTTTCAGAACTTC	TGAGAGGTGGTGTAAGCCATGC

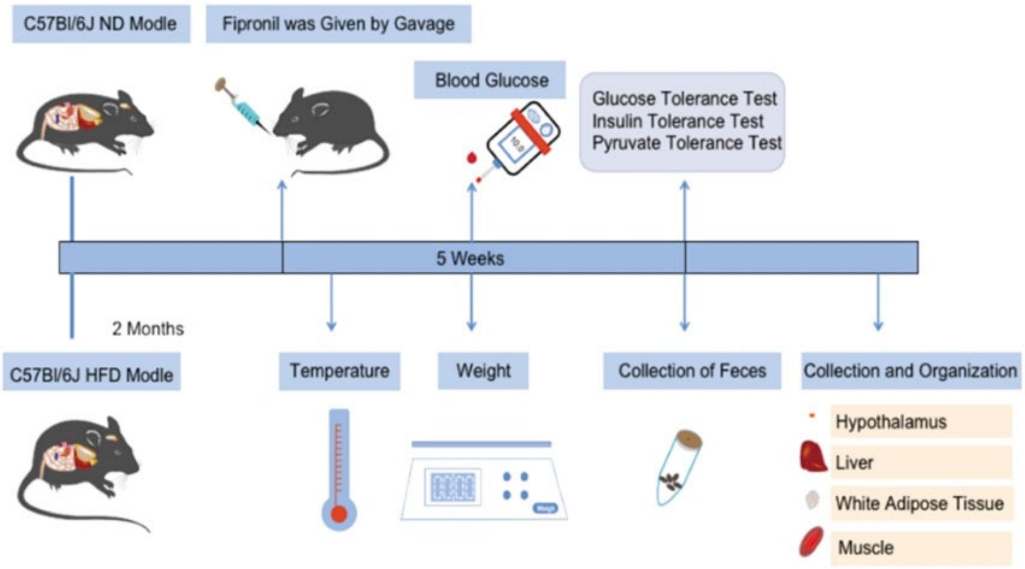

Figure S1. Experiment Design Flowchart. Fifty-six C57Bl/6j mice were divided into normal diet and high-fat diet groups. Different concentrations of fipronil experimental groups (0, 0.25 mg/kg, 1 mg/kg, 4 mg/kg) were administered orally every day. The exposure lasted 35 days, and changes in body weight, temperature, and blood glucose were recorded every week. After exposure, the glucose metabolism (systemic insulin sensitivity, liver of gluconeogenesis and glycogen, white adipose tissue, muscle, and other tissues of the insulin signaling pathway) of mice in different diet groups were detected. Feces collection was performed after 5 weeks of fipronil gavage, and hypothalamus, liver, white adipose tissue, and muscle were collected.
